# Results of 20 consecutive patients treated with the Repiphysis expandable prosthesis for primary malignant bone

**DOI:** 10.1186/s40064-015-1582-6

**Published:** 2015-12-22

**Authors:** Joseph Benevenia, Francis Patterson, Kathleen Beebe, Kimberly Tucker, Jeffrey Moore, Joseph Ippolito, Steven Rivero

**Affiliations:** Department of Orthopaedic Surgery, Rutgers New Jersey Medical School, 140 Bergen Street, ACC Building, Suite D-1610, Newark, NJ 07103 USA

**Keywords:** Orthopaedic surgery, Musculoskeletal oncology, Expandable endoprosthesis

## Abstract

Limb-salvage for primary malignant bone tumors in pediatric patients presents a unique challenge when resection includes an active physis. Early expandable prostheses required open surgical procedures to achieve lengthening. Newer prostheses are capable of achieving expansion without open procedures through the use of an electromagnetic field. This study reports our results with 90 consecutive expansion procedures using the Repiphysis^®^ prosthesis. We retrospectively reviewed the records of 20 patients (22 limbs) who underwent limb-salvage using the Repiphysis^®^ prosthesis from 2003 to 2015. There were 9 males and 11 females with a mean age of 9 years and 9 months (6–16 years). Reconstruction included the distal femur in 11 cases, total femur in four, proximal tibia in three, proximal humerus in three, and total humerus in one. Complications were reviewed and functional scores were recorded using the MSTS/ISOLS system. Five patients had a second prosthesis implanted during the course of the study for a total of 27 prostheses. The mean follow-up was 57 (6–148) months. Four patients have not been expanded: three due to death prior to lengthening, and one patient who has not yet developed a leg length discrepancy. Ninety consecutive expansion procedures were performed in 18 limbs in 16 patients. A mean of 9 (5–20) mm was gained per expansion and 4.8 cm per patient who has undergone expansion to date. Seven patients have reached skeletal maturity and have been converted to an adult endoprosthesis. These patients averaged 8 expansions per patient and a mean of 7.4 (1.8–12.9) cm in length gained. There were 15 complications in 11 patients including one dislocation, one contracture, four cases of aseptic loosening, five structural failures (three expansion mechanism failures and two tibial fractures), three deep infections, and one case of local recurrence. The mean MSTS score was 80 % (37–97 %) and the limb retention rate was 95 %. The results of this study are comparable to previous studies involving non-invasive prostheses. This study hopefully provides additional data for clinicians to consider when faced with limb threatening sarcomas in the immature skeleton.

## Background

Limb salvage became popularized following detailed reports by Mankin and Marcove in 1976 (Mankin et al. 1976; Marcove 1976). These papers, in conjunction with the classic report by Simon and Rougraff showing no survival or local control advantage to amputation, paved the way for limb preservation procedures (Rougraff et al. 1994).

Limb-salvage in pediatric patients poses an additional challenge compared to adults due to the presence of an open physis. In order to obtain a wide surgical margin, the growing physis must often be removed, creating a limb-length discrepancy. Lewis published one of the first reports on the use of expandable endoprostheses to help minimize limb length discrepancy (Lewis 1986; Kenan and Lewis 1991). These designs incorporated the use of a screw extension mechanism, where a chuck key would be inserted through an incision to expand the length of the prosthesis and thus the extremity, while later methods utilized modular body segments to achieve lengthening, requiring open surgical procedures (Lewis 1986; Kenan and Lewis 1991; Eckardt et al. 2000; Neel and Letson 2001). Jeys reported on periprosthetic infections in orthopaedic oncology conditions and found the use of expandable prostheses in children as a significant risk factor with a rate of 18 %, (p = 0.007), and that infection was increased as much as 5 % per lengthening procedure (Jeys et al. 2005). In order to accurately address limb surgery in children with sarcomas the authors feel they should mention two other techniques, amputation and arthroplasty. The former has few surgical side effects with decreased functional scores (Eckardt et al. 1985; Simon et al. 1986). Rotationplasty in the growing child has excellent function with few surgical complications (Hardes et al. 2003). Acceptance of the physical appearance of this option has limited its use (Veenstra et al. 2000).

The Repiphysis^®^ non-invasive expandable prosthesis (Microport Orthopedics, Arlington, Tennessee, USA, originally manufactured as the Phenix prosthesis, Phenix Medical, Paris, France) was the first endoprosthesis design to introduce the concept of a non-invasive expansion procedure involving the use of an electromagnetic field (Wilkins and Soubeiran 2001; Neel et al. 2003; Gupta et al. 2006). This implant allows for a serial number of lengthenings to be performed without an open surgical procedure. The closed lengthenings avoid incisions. This in turn avoids all those problems associated with open surgery, blood loss and acute lengthenings. Acute lengthenings with sudden elongation, may produce neuropathy, vascular compromise, and contracture (Herzog and Hefti 1992; Janovec and Polách 1990) The aim of the current study is to report our experience with 20 patients treated with the Repiphysis^®^ non-invasive expandable prosthesis for limb-salvage.

## Methods

We retrospectively reviewed the records of 20 consecutive patients who underwent implantation of the Repiphysis^®^ noninvasive expandable prosthesis at our institution from 2003 to 2015. Minimum follow-up was 14 months unless patients died of disease or the implant failed prior. We have previously reported on early results of the first 12 of these patients and present them with longer term follow-up and additional lengthening procedures (Beebe et al. 2010) in addition to eight new patients. Data collected included patient demographics, pathology, lengthening, total expansion length, complications, and functional status. Implantation of the Repiphysis^®^ prosthesis is indicated for limb-salvage in skeletally immature patients in which wide resection includes removal of an active physis and the patient is left with a projected limb-length discrepancy of ≥6 cm (Harvey et al. 2010; Holm et al. 1994; Papaioannou et al. 1982; Song et al. 1997; Stanitski 1999). Growth remaining was determined according to the standard methods after bone age was assessed (Anderson et al. 1963; Dimeglio 2001). For use in the humerus, the Repiphysis^®^ was discussed with the patient and family as a limb-salvage option and Compassionate Use Guidelines were followed with our Institutional Review Board (IRB) permission. Patients were indicated for expansion when chemotherapy was complete and a limb-length discrepancy of ≥1 cm was noted clinically. Functional scores were recorded using the MSTS/ISOLS functional scoring system (Enneking et al. 1993). Scores were obtained upon completion of rehabilitative therapy, or at most recent follow-up if therapy was not completed.

**Fig. 1:**
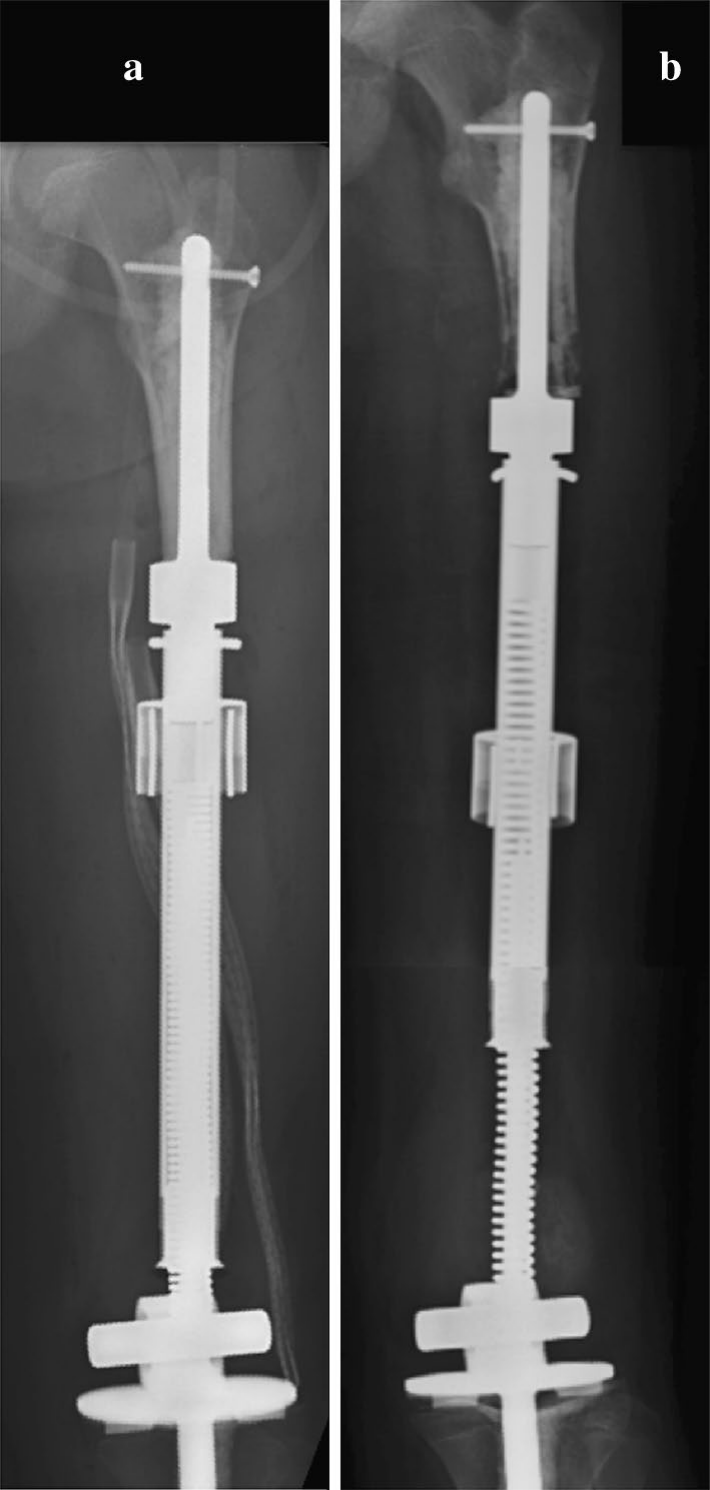
Patient with a femoral prosthesis prior to undergoing any expansion procedures (**a**) and at the maximum growth potential for the implant (**b**)

When the prosthesis is exposed to an external ring producing an electromagnetic field, the heat softens the outer polyethylene cylinder, thus unlocking the flared trumpet and permitting the prosthesis to expand as the spring decompresses and allows the inner tube to slide within the polyethylene cylinder (Wilkins and Soubeiran 2001) All expansions are performed with conscious sedation or general anesthesia in the operating room with fluoroscopic guidance, using a C-arm, radiolucent table, radiolucent ruler, and goniometer. Expansion procedures typically take 20 min. The radiofrequency signal is applied in pre-set “pulses” under fluoroscopic guidance until the desired lengthening is achieved. Upon conclusion of the lengthening, the metallic trumpet cools down and locks the prosthesis back in place in a new portion of the polyethylene cylinder (Fig. 1a, b).

## Results

The results are summarized in Table 1. Twenty patients had 22 sites treated with 27 non-invasive expandable endoprostheses from 2003 to 2015. Two patients (16 and 20) had a second osteosarcoma: 1 in the contralateral femur, 1 in the ipisilateral humerus. The diagnosis was osteosarcoma in 18 patients and Ewing’s sarcoma in two. There were 9 males and 11 females studied with a mean age of 9 years and 9 months (6–16 years) at the index procedure and a mean follow-up of 57 (6–148) months, with an endpoint of death or latest follow-up. Of 22 limbs in 20 patients, 17 were treated at the author’s institution primarily with Repiphysis. Two patients (14 and 17) were treated at outside institutions and presented to our institution after a failed implant. One patient had an earlier invasive expandable prosthesis prior to the availability of Repiphysis, which was explanted following deep infection from an open lengthening. This patient then had a Repiphysis implanted following eradication of infection (patient 7). One patient initially had an intercalary allograft, which fractured and was revised with a Repiphysis (patient 16a).

**Table 1:** Overview of patient characteristics, lengthening, and complications

Pt.	Age at Initial surgery (Y + M)	Diagnosis	Stage	Location of lesion	No. of exp	Length gained (cm)	Follow-up (months)	Converted to adult prosthesis?	Type of failure	Onco. status	Complications	MSTS score (%)
1	9 + 8	Osteosarcoma	IIB	R Prox Tibia	2	1.8	144	Yes	II	CDF	Aseptic loosening	93
2	9 + 10	Osteosarcoma	IIB	R Dist Femur	10	10	148	Yes	II, III	NED	Aseptic loosening and tibial fracture	77
3	9 + 11	Osteosarcoma	IIB	R Dist Femur	1	2	22	No		DOD		67
4	8 + 10	Osteosarcoma	IIB	L Prox Humerus	6	5.8	116	Yes		CDF		93
5	11 + 2	Ewing’s Sarcoma	IIB	L Dist Femur	8	7.9	137	Yes	I, IV	CDF	Contracture, infection	97
6	10 + 8	Osteosarcoma	IIB	L Dist Femur	6	7	61	No		CDF		77
7	16	Ewing’s Sarcoma	IIB	L Prox Humerus	6	5.5	75	No	I	CDF	Contracture/dislocation	93
8	7 + 9	Osteosarcoma	IIB	L Dist Femur	0	0	10	No		DOD		70
*9* ^A^	12 + 1	Osteosarcoma	IIB	L Prox Tibia	5	4.5	64	No	III × 2	DOD	Prosthesis failure, tibial fracture	93
10	10 + 6	Osteosarcoma	III	L Dist Femur	2	2.2	27	No		DOD		80
11	12 + 9	Osteosarcoma	IIB	L Dist Femur	0	0	12	No	V	DOD	Hip disarticulation	80
12	7	Osteosarcoma	IIB	L Prox Humerus	8	6.9	71	Yes	III	CDF		87
13^A^	9 + 7	Osteosarcoma	IIB	L Dist Femur	4	4	41	No	IV	DOD	Infection	63
14^A,C^	9 + 11	Osteosarcoma	IIB	L Dist Femur	9	6.8	67	Yes	III, IV	CDF	Prosthesis failure, Infection	87
15^A^	9 + 4	Osteosarcoma	IIB	L Dist Femur	14	12.9	63	Yes	II	CDF	Aseptic loosening	90
16a^A^	9 + 2	Osteosarcoma	IIB	R Dist Femur	1	1	54	No	II	CDF	Aseptic loosening	93
16b	11	Osteosarcoma	IIB	L Dist Femur	1	1	30	No		CDF		83
17^C^	12	Osteosarcoma	IIB	L Dist Femur	0	0	6	No		DOD		47
18	6	Osteosarcoma	IIB	R Dist Femur	2	2	30	No		CDF		90
19	7 + 1	Osteosarcoma	IIB	L Prox Tibia	0	0	14	No		CDF		37
20a	6 + 9	Osteosarcoma	IIB	R Dist Femur	3	2.3	37	No		AWD		83
20b^B^	7 + 6	Osteosarcoma	III	R Prox Humerus	2	1.7	17	No		AWD		80

Types of failure: soft-tissue (I), aseptic loosening (II), structural failure (III), infection (IV), tumor progression (V)

Patients 1–12 were reported on in previous study

Patients 16 and 20 had diagnosis of Osteosarcoma at two different sites

*DOD* dead of disease, *NED* no evidence of disease, *AWD* alive with disease, *CDF* continuously disease free

^A^Indicates that the patient had second Repiphysis expandable prostheses at same site

^B^Patient presented with a second bone lesion without pulmonary metastasis

^C^Indicates patients initially treated at an outside institution, and presented to our institution after failed implant

Reconstruction included the distal femur in 11 cases, total femur in four, proximal tibia in three, proximal humerus in three, and total humerus in one. Five patients had a second endoprosthesis implanted during the study period for a total of 27 non-invasive expandable prostheses in our 20 patient cohort. Of these five second implants, one was for a deep infection in which the original Repiphysis^®^ was removed and a new one re-implanted after eradication of the infection (Patient 13), and four implants were exchanged for a new prosthesis after they had reached their maximum elongation to permit continued expansion (Patients 9, 14, 15 and 16a). Generally the authors waited until the patient required an expansion or the implant became symptomatic before explant and re-implantation of a second new prosthesis.

At the time of latest follow-up, 65 % of patients are alive (11 CDF, 7 DOD, 1 AWD, 1 NED). Ninety consecutive expansion procedures were performed in 18 limbs in 16 patients. The patients who have not had any expansions include three patients who died of disease prior to lengthening (Patients 8, 11 and 17) and one patient who has not yet established a limb-length discrepancy warranting expansion (Patient 19). Two patients (14, 15) had a second prosthesis that underwent additional expansion. A mean of 9 (5–20) mm was gained per expansion and 4.8 cm per patient who has undergone expansion to date.

### Conversion to adult implants

**Fig. 2:**
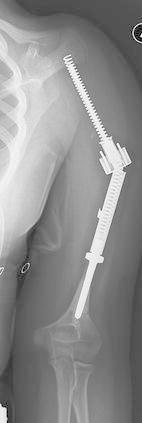
Breakage of the expansion mechanism in a proximal humeral prosthesis

Among the prostheses which have undergone expansion: eight are still undergoing expansion procedures, three patients have died of disease prior to reaching skeletal maturity (Patients 3, 10 and 13), and seven patients have reached skeletal maturity and been converted to a non-expandable adult endoprosthesis (Patients 1, 2, 4, 5, 12, 14, and 15) (Fig. 1c). Among those who have reached skeletal maturity, there were an average of 8 expansions per patient and a mean length gained of 7.4 (1.8–12.9) cm. Six of the seven patients have no limb length discrepancy. The seventh patient had a proximal humerus replacement, which experienced an expansion mechanism failure (Fig. 2). Because the patient was nearing skeletal maturity, revision included conversion to an adult endoprosthesis. The patient has a 2.5 cm limb length discrepancy, which is not clinically symptomatic, with an MSTS score of 87 % (Patient 12).

**Fig. 3:**
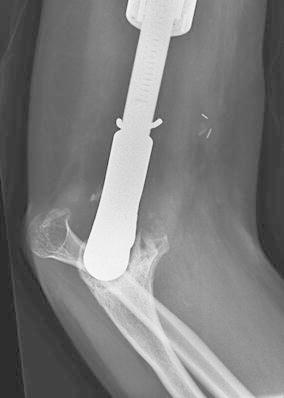
A contracture/dislocation in a patient with a humeral endoprosthesis

**Fig. 4:**
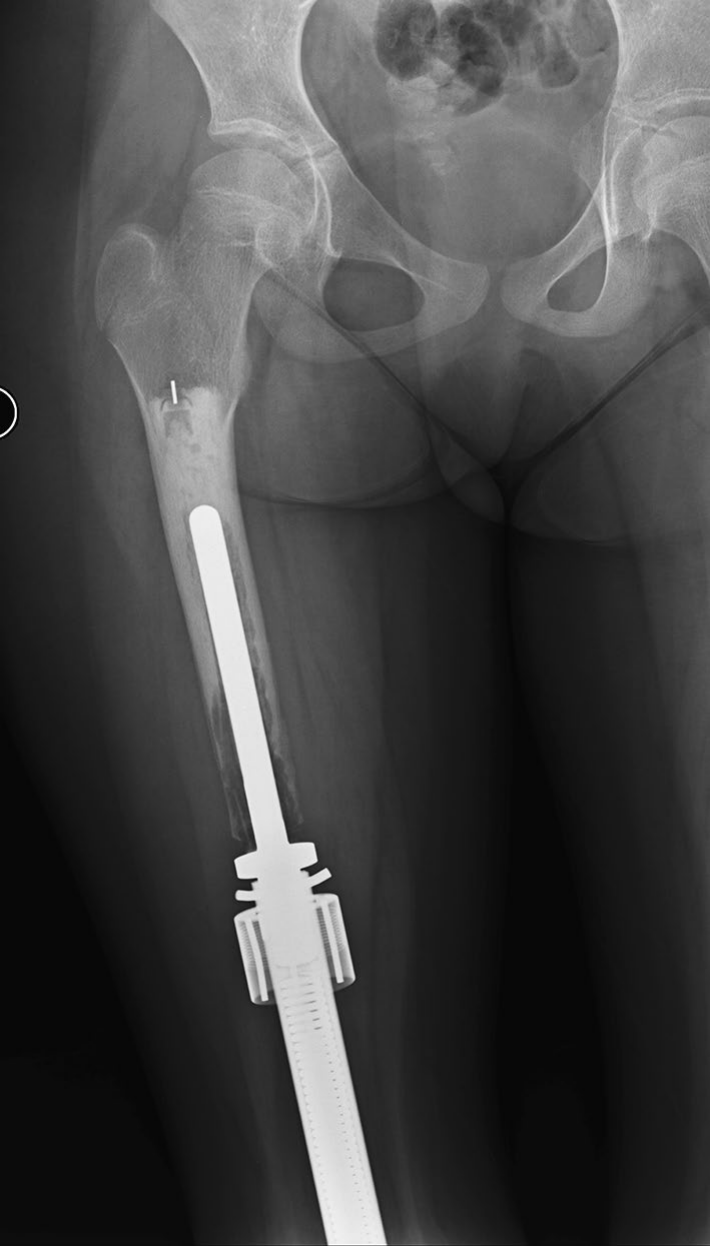
Aseptic loosening of the proximal stem in a femoral endoprosthesis

**Fig. 5:**
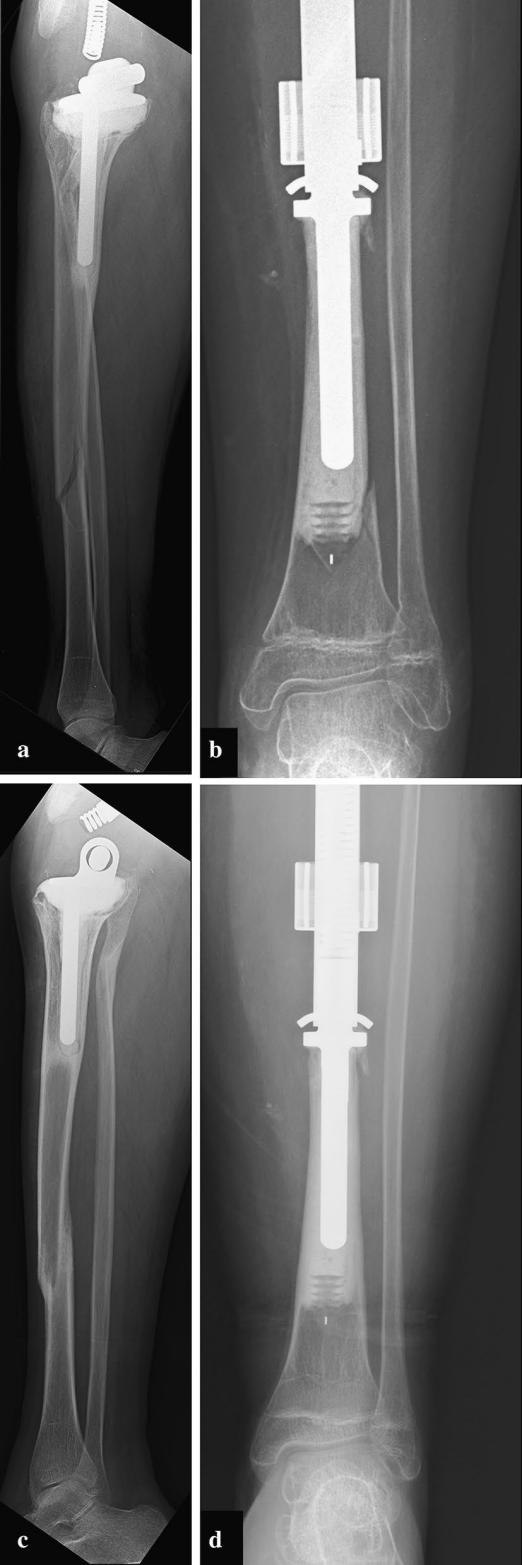
Periprosthetic tibial fractures in patients with a distal femoral prosthesis (**a**) and a proximal tibial prosthesis (**b**) which healed with non-operative management (**c**, **d**)

**Fig. 6:**
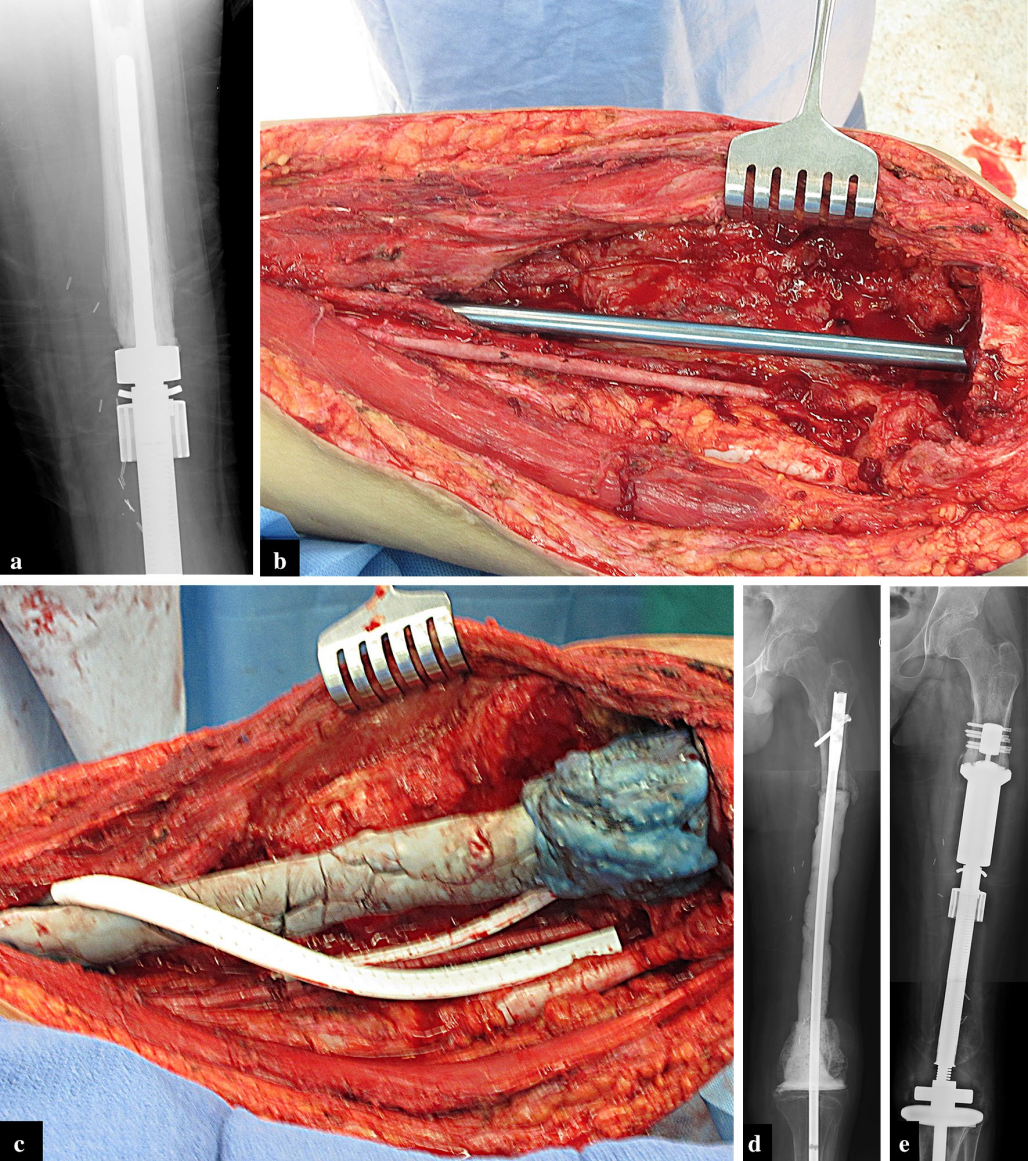
**a** Radiographic evidence of septic loosening of a femoral prosthesis. **b**, **c** Removal of the implant and placement of an antibiotic spacer consisting of an intramedullary nail (**b**) covered in antibiotic cement (**c**). **d** A radiograph of the antibiotic spacer after removal of the prosthesis. **e** Reimplantation of the prosthesis after eradication of the infection

During the course of our study period we experienced 15 complications (56 %). We classified our complications according to the five modes of failure for tumor endoprostheses (Henderson et al. 2011; Palumbo et al. 2011) (Table 2). There were two type I (soft-tissue) failures. A patient with a distal femoral prosthesis underwent an arthroscopic release of the quadriceps due to an extensor mechanism contracture (Patient 5). Another patient had initially presented with a custom expandable humeral prosthesis that became infected and was treated with an explant, IV antibiotics, and a spacer followed by re-implantation using a Repiphysis^®^. The Repiphysis^®^ elbow became stiff after six lengthenings, with a flexion contracture and a proximal radial-ulnar joint dislocation resulting from previous proximal ulnar physeal arrest, secondary to infection (Patient 7, Fig. 3). There were four type II (aseptic loosening) failures in which there was evidence of loosening of the stem of the prosthesis, occurring at a mean of 50.5 (23–83) months after initial surgery (Patients 1, 2, 15 and 16a, Fig. 4). Two of these were at skeletal maturity and revised with an adult prosthesis, and the other two were revised with a second Repiphysis^®^. Five patients experienced a type III (structural) failure. There were three failures of the expansion mechanism of the prosthesis (Patients 9, 12 and 14). Patients 9 and 12 were converted to an adult prosthesis and patient 14 was revised to a second Repiphysis^®^. The other two type III failures were periprosthetic tibial fractures, which were managed non-operatively (Patients 2 and 9, Fig. 5a–d). Three patients experienced a type IV (infection) failure (Patients 5, 13, and 14, Fig. 6a). These were treated by removal of the implant and placement of a custom antibiotic spacer (Fig. 6b–d), IV antibiotics for six weeks, then oral antibiotics for an additional six weeks, with subsequent re-implantation upon eradication of the infection. One of these patients had a second Repiphysis^®^ re-implanted (Fig. 6e), and the other two were converted to an adult prosthesis. All of these patients were able to achieve limb length equality. Finally, one patient who did not have any expansions experienced a type-V (tumor progression) failure and required a hip disarticulation (Patient 11). The overall limb preservation rate in our patients was 95 % (21/22 limbs). The mean MSTS score was 80 % (37–97 %).

**Table 2:** Five modes of tumor prosthesis failure according to Henderson et al. (2011, 2012)

Type of failure	Description	No. of pts
I	Soft-tissue: A functional deficiency of the soft tissue attachments about the implant. Includes instability, tendon rupture, and wound dehiscence	2
II	Aseptic loosening: Clinical and radiographic evidence of loosening of the prosthesis	4
III	Structural failure: Failure of either the implant or surrounding bone. Includes fractures of the prosthesis, periprosthetic fractures, and a deficient bony supporting structure	5
IV	Infection: Deep infection requiring removal of the implant	3
V	Tumor progression: Re-operation due to local tumor recurrence or metastatic disease progression	1

## Discussion

Over the past 30 years there have been several expandable implant systems designed to be used in the growing child. In the 1990’s, new prostheses were developed that allowed non-invasive lengthening through the application of an external electromagnetic field (Wilkins and Soubeiran 2001; Neel et al. 2003; Baumgart et al. 1997).

The Repiphysis^®^ prosthesis, originally manufactured as the Phenix^®^ prosthesis in France, was the first of these non-invasive expandable implants used in the United States. Early experience with the prosthesis was reported by Wilkins and Souberian (Wilkins and Soubeiran 2001), who examined the functional outcomes after 21 lengthenings in six patients. One of the major advantages of the Repiphysis^®^ over earlier designs is that the non-invasive nature of the expansion procedure permits frequent expansions of smaller increments with minimal patient discomfort. This decreases the risk of stretch injury to nerves and blood vessels and resection of soft tissue and pseudocapsule around the prosthesis. It avoids an open surgical procedure each time expansion is necessary. Although there were no complications attributed to lengthening procedures themselves, Wilkins and Souberian reported two mechanical complications in one patient (Wilkins and Soubeiran 2001).

In 2003, a multicenter study by Neel et al. (Neel et al. 2003) reported the results of 60 expansions in 18 Repiphysis^®^ prostheses implanted in 15 patients. Mean follow-up was 21.5 (12–33) months. There were no complications associated with lengthening procedures themselves. They had a mean of 3.7 cm of lengthening and MSTS scores averaging 90 %, but the prostheses were subject to a 44 % complication rate including six prosthesis fractures and two aseptic loosenings (Neel et al. 2003). Longer-term studies using the Repiphysis^®^ prosthesis have produced similar results, with mean MSTS scores ranging from 67 to 90 % and patients gaining a mean of 2.5–3.9 cm of lengthening (Saghieh et al. 2010; Cipriano et al. 2015).

The Stanmore Juvenile Tumour System (JTS, Stanmore Implants Worldwide, Stanmore, UK) also incorporates a magnetic field for non-invasive expansion after tumor resection in skeletally immature patients. In contrast to the Repiphysis^®^ design, the JTS contains a magnetic disc, a gearbox, and a power screw embedded within the shaft of the prosthesis. When exposed to an external electromagnetic field the implant is expanded at a fixed rate. One of the limitations of the JTS is that the use of MRI in these patients is contraindicated due to interference with the electromagnetic motor (Gupta et al. 2006).

Hwang et al. reported on the use of the JTS in a series of 25 patients published in 2012 (Hwang et al. 2012). They reported a mean of 3.2 cm of lengthening with a mean MSTS score of 85 % and a complication rate of 38 %. Other studies have reported similar experience with the JTS, with a mean 3.7–4.5 cm of lengthening, MSTS scores ranging from 82 to 87 %, and follow-up of 41.2 (22–104) and 48 (23–146) months (Picardo et al. 2012; Henderson et al. 2012).

The current study reports the results of 20 pediatric patients treated at 22 sites with 27 Repiphysis^®^ prostheses with a mean follow-up of 57 months. This is subject to all the inherent limitations of a retrospective study design. The results are comparable to previously published studies involving the Repiphysis^®^ in terms of both function and complications. The overall limb-salvage rate was 95 %, with one patient requiring a hip disarticulation for an oncologic complication (local recurrence). On average, patients gained 9 mm of length per expansion and 4.6 cm per patient who has undergone lengthening. So far, those that reached skeletal maturity were lengthened by a mean of 7.4 cm. To the author’s knowledge, there has been no other non-invasive study with this amount of expansion and only one other series with greater reported mean expansion which used open lengthening techniques (Schiller et al. 1995). This is the largest study cohort of Repiphysis among similar published studies (Beebe et al. 2010; Cipriano et al. 2015; Gupta et al. 2006; Neel et al. 2003; Saghieh et al. 2010; Wilkins and Soubeiran 2001).

The mean MSTS score in this study was 80 %, which falls between the 67 % and 90 % MSTS scores reported in previous studies. There was only one case of limb length discrepancy, 2.5 cm limb length discrepancy in the humerus of patient 12. The complication rate was 56 % (15 complications in 27 prostheses); 2 type I, 4 type II, 5 type III, 3 type IV, and 1 type V. Our infection rate is comparable to previously published studies involving Repiphysis^®^ and with both traditional invasively expandable endoprostheses, which have demonstrated an infection rate up to 18 % (Wilkins and Soubeiran 2001; Saghieh et al. 2010; Cipriano et al. 2015; Hwang et al. 2012; Picardo et al. 2012).

A limitation of the Repiphysis^®^ relative to the JTS is that the Repiphysis^®^ requires conversion to a non-expandable adult prosthesis at skeletal maturity while the JTS is designed to be a permanent implant. A limitation of the JTS relative to the Repiphysis^®^ is that patients are unable to undergo an MRI. Given the value of MRI for re-staging in this patient population, the use of devices that prohibit this imaging modality must be carefully considered (Heindel et al. 2014; Nascimento et al. 2014). Furthermore, it is not known whether a patient will be able to function with a pediatric size endoprosthesis in the long-term.

In conclusion, the Repiphysis^®^ prosthesis (Microport Orthopedics, Arlington, Tennessee, USA) is an option for limb-salvage in skeletally immature patients and has similar drawbacks to other designs. It requires conversion to an adult prosthesis with a limb retention rate of 95 % and has a mean lengthening of 7.4 cm at maturity. Given the complications associated with the use of this prosthesis, as well as other expandables, the authors caution against uninformed use and recommend full and complete disclosure of all other treatment options with patients and their families before surgery.
